# Event Detection for Distributed Acoustic Sensing: Combining Knowledge-Based, Classical Machine Learning, and Deep Learning Approaches

**DOI:** 10.3390/s21227527

**Published:** 2021-11-12

**Authors:** Mugdim Bublin

**Affiliations:** FH Campus Wien, University of Applied Sciences, 1100 Vienna, Austria; mugdim.bublin@fh-campuswien.ac.at; Tel.: +43-1-606-68-77-2133

**Keywords:** deep neural networks, Distributed Acoustic Sensing, machine learning, signal processing

## Abstract

Distributed Acoustic Sensing (DAS) is a promising new technology for pipeline monitoring and protection. However, a big challenge is distinguishing between relevant events, like intrusion by an excavator near the pipeline, and interference, like land machines. This paper investigates whether it is possible to achieve adequate detection accuracy with classic machine learning algorithms using simulations and real system implementation. Then, we compare classical machine learning with a deep learning approach and analyze the advantages and disadvantages of both approaches. Although acceptable performance can be achieved with both approaches, preliminary results show that deep learning is the more promising approach, eliminating the need for laborious feature extraction and offering a six times lower event detection delay and twelve times lower execution time. However, we achieved the best results by combining deep learning with the knowledge-based and classical machine learning approaches. At the end of this manuscript, we propose general guidelines for efficient system design combining knowledge-based, classical machine learning, and deep learning approaches.

## 1. Introduction

**Figure 1:**
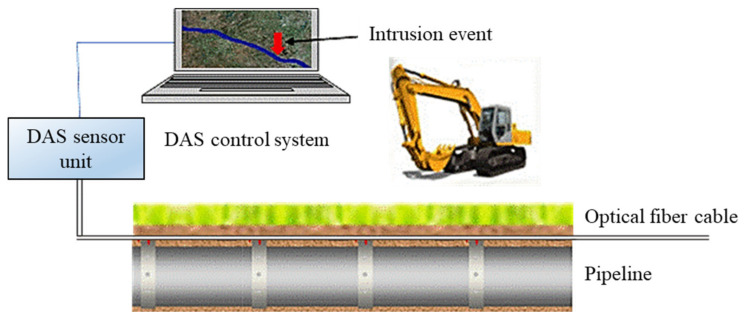
Distributed acoustic–optical sensors for pipeline monitoring.

Pipeline accidents could cause human deaths, serious injuries, and material costs [1]. Presently, pipeline monitoring and protection is often performed by expensive and laborious physical inspection methods such as helicopter or vehicle patrols [2]. Distributed acoustic-optical sensors (DAS) are a comparatively new approach (see Figure 1). Such systems permit the supervision of long-distance pipelines using fiber optics [3,4,5,6,7,8,9,10].

Since fiber optic cables are usually laid along the modern pipelines, no additional investment in equipment installation is needed. However, the application of distributed acoustic–optical sensors requires advanced computer algorithms capable of recognizing events of interest such as manual and/or excavator digging, tapping, and intrusion detection. The design of efficient algorithms for event detection based on DAS measurements is especially hard due to a huge variety of scenarios such as various kinds of excavators and soil types, different temperature and weather conditions, as well as possible interference by other signal sources such as highways, railways, wind turbines, and agricultural machines, etc. Signal processing [11] and machine learning (ML) [12,13,14,15,16,17,18,19,20,21] are promising approaches for tackling such problems.

Most of the current investigation and available literature focuses on the extracting of the signal from the fiber, which is completed by interferometer assemblies. In [3], the sensing of vibration using an optical frequency-domain reflectometry (OFDR) technique was demonstrated in a 17-m length fiber up to a frequency of 32 Hz. A Mach–Zender interferometer was used together with standard single-mode fibers. However, the work focuses on the extraction of the signal, and the classification of events from the signal is not discussed. Juarez et al. [4] describe the OTDR system for the detecting of intruders in laboratory and field tests using a 12-km-length fiber. In the field, tests successfully demonstrated the ability to detect an 80-kg person walking over the fiber. However, the ability to classify different events has not been investigated. Choi [7] reports on the use of OTDR for intruder detection over a cable buried 30 cm deep in the ground and with a length of two km, tested with a 60-kg person walking up to a 1-m distance from the cable. Again, any change in the signal was interpreted as evidence for the intrusion event without discussing the possibility of classifying events by careful analysis of the signal signature. Kumagai [8] describes an approach to distinguish intruders climbing a fence from fence vibration due to wind to minimize false alarms. The signal of a Sagnac-type interferometer was used to classify the event using FFT frequency analysis in the 0 to 250 Hz range. It was demonstrated that the event of a person climbing the fence can be distinguished from the wind-induced fence vibration, and the resulting false alarm rate was around one per month during a 1-year test period. About two real events per day have been detected with a 100% detection rate. US patent 5,194,847 [9] discloses a method and apparatus for intrusion detection based on the OTDR technique. The details of optical setup and pulse forming as well as signal processing for the detection and location of the intruder from the backscattered signal of an interrogating pulse are described, but the classification of the event is not disclosed. Harman discloses a method and apparatus in PCT patent application WO2013/185208 [10] for short-range perimeter surveillance with two back-to-back Michelson interferometers using a cable comprising of four optical fibers. He targets to achieve a competitive price for a security installation using this arrangement and describes the necessary signal processing and post-processing techniques to extract an intruder’s location from the optical signal. Again, signal processing for the classification of the event was not disclosed.

In spite of more works on algorithms for event detection in recent times [22,23,24,25,26,27,28,29,30], research and development of such algorithms are still challenging. Although there are some applications of either classical machine learning [22,23] or deep learning (DL) [24,25,26,27,28,29,30] approaches in event detection in DAS, to our knowledge, there is not much research comparing and combining these two approaches, as can be seen in a recent review [30]. Especially, little attention is devoted to the role of human knowledge and its iterative development during the whole process. Classifiers presented in [22,23,24,25,26,27,28,29,30] are mostly multi-event classifiers able to detect different events like big/small machine hitting, pneumatic hammer, and a plate compactor, where our classifiers distinguish only between two events: ‘excavator digging’ and ‘no excavator digging’. However, our classifier achieves higher classification accuracy than most of the classifiers presented in the literature. We also provide real-time system deployment results with a high true alarm and low false alarm rate, whereas most of the references provide only the classification accuracy for different events, but not the results of the real-time system deployment. Like the references [22,23,24,25,26,27,28,29,30], we investigated supervised classifiers, both classical machine learning as well as deep learning classifiers, where for each training instance the label is provided to which class the instance belongs. Performance comparisons between our system and the systems in the references [22,23,24,25,26,27,28,29,30] are further discussed in the Results section.

In this work, we present the application of signal processing and machine learning algorithms for event detection using DAS signals generated along a pipeline. We implemented, compared, and combined two approaches for event detection: the classic machine learning approach and the approach based on and deep learning. We especially emphasize the role of human knowledge in all phases of development, from data collection through an algorithms’ selection to system evaluation. We explicitly list design decisions made according to the available knowledge at different phases of the development and outline a proposal for the whole design process.

In the sequel of the work, we first provide a short description of DAS technology. Then, we describe some machine learning and signal-processing algorithms that can be used for event detection using DAS signals. Finally, we present and discuss results and provide suggestions for further work.

## 2. Materials and Methods

### 2.1. Distributed Acoustic-Optical Sensors

**Figure 2:**
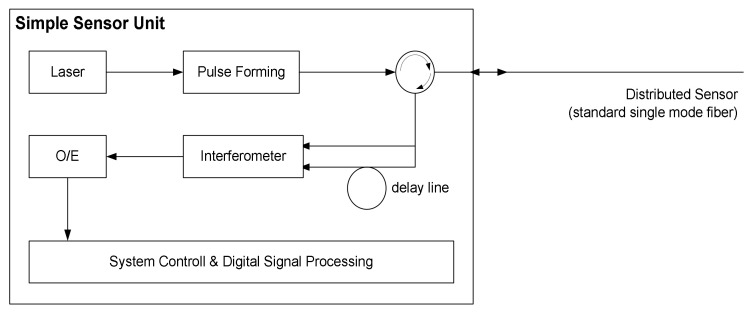
Distributed Acoustic Sensor (DAS) Unit.

Optical Time-Domain Reflectometry (OTDR) is a well-established technique used to check long-haul fiber optical connections for telecommunication. This technology is based on emitting short pulses into the fiber and recording the intensity of light reflected to the sender by Rayleigh reflection [3,4,5,6]. A distributed acoustic–optical sensor can be constructed by exploiting the fact that the refractive index of glass fiber is slightly affected by any applied pressure, including sound pressure: short pulses are emitted as in the case of OTDR, but instead, the signal intensity evaluated in the phase of the Rayleigh reflected optical signal [3,4,5,6]. The laser pulse is launched into the fiber through pulse-forming unit (see Figure 2). Rayleigh backscattered light from different positions along the fiber is then captured in the reflected signal. Finally, the signal at different positions on the fiber is determined by an interferometer as the difference between the phases of the transmitted pulse and the reflected pulses from that fiber position (Φ-OTDR). Delay line brings light reflected at short distances—say, 10 m apart—to the interferometer at the same time. Alternatively, no delay line is needed if the pulse-forming unit can create two short pulses emitted in the fiber at, e.g., 20 m.

The optical/electrical (O/E) unit performs the conversion of the optical to the electrical signal. By evaluating the interferometer every 100 nanoseconds, this procedure captures the sound pressure every ~10 m. By emitting pulses at a rate of, e.g., 1000 Hz, one obtains a distributed acoustic–optical sensor that is capable of detecting the vibrations’ pressure up to distances of 40 km at regular distance of 10 m and up to frequencies of 500 Hz. Optical fiber sensors have certain advantages that include immunity to electromagnetic interference, lightweight, small size, high sensitivity, large bandwidth, and ease in implementation, as fiber cables are often already installed for communication purposes in critical infrastructures. Strain, temperature, and pressure are the most widely studied signals used in optical fiber sensing [5,6]. The tiny pressure change induced by acoustic events in the surrounding of an optical fiber can be measured by optical means over large distance, and therefore, surveillance of large areas becomes possible using this technology

We used DAS based on Φ-OTDR. In our DAS system, polarization phase changes of the Rayleigh backscattering light at different sections of the fiber (each 10 m) are sampled at the rate of 2 kHz and provided each second as the input for the ML algorithms. The ML performs classification decisions (‘excavator’ no ‘excavator’). These decisions are then evaluated by the heuristic rules derived from the human domain knowledge before an alarm is eventually raised.

The system was deployed on Windows operating system using GNU C for extracting signals from the reflected light and doing basic preprocessing. For further signal processing and machine learning, we used Matlab and scikit-learn library. Details of the system used are provided in Table 1.

### 2.2. Signal Processing and Machine Learning for DAS Event Detection

While the classical literature describes basic signal processing to resolve an event from the OTDR signal and thereby generate alarms [7,8,9,10], the classification of event types from the signal is a modern research topic [22,23,24,25,26,27,28,29,30]. There is a need to monitor and detect specific, safety-relevant events with a low false alarm rate among many ‘harmless’ background events. Although it is relatively new research area requiring multidisciplinary approach, we are standing on the ‘shoulders of giants’ and can exploit a vast body of knowledge in signal processing and pattern recognition in general [11,12,13] and more recent advances like deep learning specifically [14,15].

**Figure 3:**
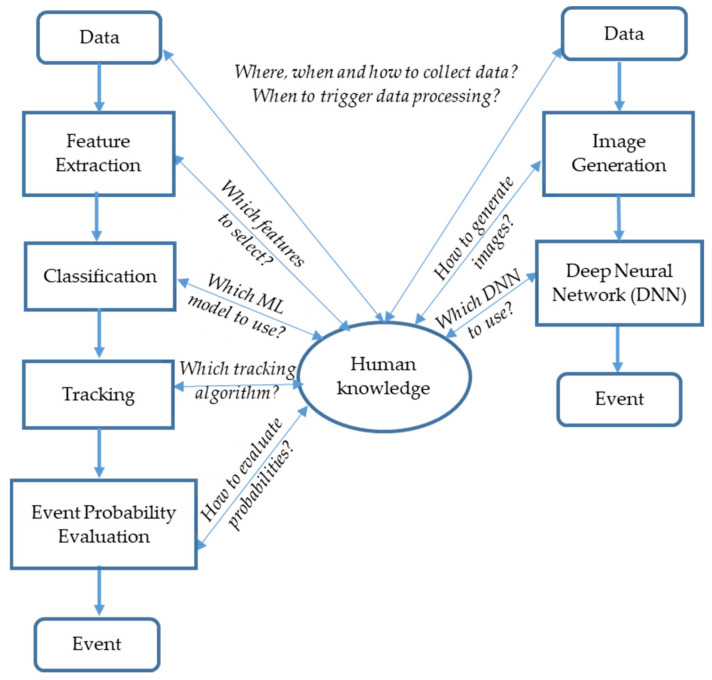
Two methods for event detection by DAS: classic machine learning approach (**left**) and deep neural networks approach (**right**). Note the central role of human knowledge.

The classical task machine learning approach consists of the following phases: data collection, data preprocessing, feature extraction, feature classification, tracking, and event probability evaluation (see Figure 3, left).

In Figure 3, we emphasize the central role of human knowledge in making design decisions about data collection strategy, feature selection, and algorithms selection for machine learning and tracking. Human knowledge comprises our whole domain and physics knowledge: how signals arise and propagate over the Earth, what possible interference sources exist, and which machine learning and tracking algorithms we can use. Human knowledge is iteratively refined and improved during the whole project. In the following, we shortly describe each of the phases using classing machine learning approach.

#### 2.2.1. Feature Selection and Extraction

The art of science of machine learning consists of selecting appropriate features and classification algorithms for the task at hand. Especially, feature selection is demanding task that requires high effort and very good domain knowledge.

Before feature extraction, standard signal-processing techniques like signal smoothing and filtering are used for data pre-processing and visualization, which are necessary prerequisites for further signal analysis and pattern recognition [11,12]. To reduce computation effort, a threshold selection can also be used to only evaluate the fiber sections with a signal above the appropriate threshold.

The feature extraction task is to define and calculate the specific characteristics of the signals (features) useful for classification [12,13]. The outputs of feature extractions are N-dimensional vectors that are mapped by feature classifier to the classes in an N-dimensional feature space. The features can be extracted from signal characteristics in time, frequency, or in time and frequency (scale) domains:

- Time features: time energy distribution (TE), principal component analysis (PCA), and correlation-based features;
- Frequency features: fast Fourier transform (FFT), short-time fast Fourier transform (STFT), and cepstral coefficients (CC);
- Time-frequency features: continuous wavelet transform (CWT), discrete wavelet transform (DWT), and wavelet packet transform (WPT).

#### 2.2.2. Feature Classification: Machine Learning Algorithms

The task of feature classifiers, i.e., machine learning algorithm, is to assign the N-dimensional feature vectors obtained by feature extraction to the different classes of events. In our case, there are two event classes: intrusion by an excavator and no excavator intrusion. In general, it means dividing N-dimensional feature space into several regions and associating the regions with the classes. In the following, we provide an overview of some popular classifiers according to [12,13,14,15] that we evaluated in our work. We used supervised machine learning, where for each training instance a label is provided to which class the instance belongs.

- Decision trees are (tree-like) graphs in which each internal node represents an “if”-test on a feature, each branch represents the outcome of the test, and each leaf node represents a class label.
- Random Forest is an ensemble of decision trees improving accuracy by combining decisions of different classifiers (decision trees).
- Support Vector Machines (SVM) separates the classes by choosing the hyperplane that maximizes the distance between the hyperplane and the closest points in each feature space region, which are called support vectors. In the cases of feature vectors that are nonlinearly separable, a kernel function maps the input vectors to a higher dimension space in which a linear hyperplane can be used to separate the vectors.
- Artificial Neural Networks (ANNs) are classifier methods that do not need special assumptions on the underlying probability models [12,13,14,15]. A classical version of the ANN is the so-called fully connected feed-forward ANN that takes features as an input layer and forward signals from the neurons of one hidden layer to the other hidden layer and finally to the output layer, which usually provides the class probabilities. ANN can learn from examples, i.e., adapt internal weights between neurons so that the predefined feature vectors are optimally allocated to the predefined classes (learning examples). Classical ANNs are fully connected feed-forward networks where each neuron in one layer is connected to all neurons of the previous layer. This kind or ANN is also called multi-layer perceptron (MLP). However, the connections between neurons in the deep learning ANN are adapted for the task at hand, e.g., CNN for image recognition use inspired from human visual system to connect neurons in distributed and hierarchical manner to build a kind of image filters [14,15].
- Deep Neural Networks: among ANN algorithms, especially deep neural networks (DNN) with many layers of neurons organized in hierarchical manner, have drawn attention in recent times due to their superb classification performance and their ability to extract features from raw data [14,15]. As input signal for event detection, an image that has been previously generated from sensor measurements by diverse image processing algorithms can be used [18,19]. DNN can then operate on images to find the DAS events. For image classification, especially effective are convolutional neural networks (CNN) that hierarchically extract features from an image [14]. For example, edges in images are extracted by a first hidden layer, shapes are then extracted in the second layers using edges from the first layer as the input, and finally, the whole objects are extracted in the last hidden layer.

Recently, deep learning is increasingly used for event detection using fiber sensing, as described in [24,25,26,27,28,29,30]. The big advantage of deep neural networks is their capability to extract the relevant features from the raw data in a hierarchical manner without need for much domain knowledge (see Figure 3, right). One also does not need to search for optimal classifiers for the selected features, since the deep neural networks offer a unified approach for feature selection and classification. Together with their superior performance, these two advantages make deep neural networks a promising approach for many machine-learning tasks.

### 2.3. Tracking and Probability Evaluation

After detecting and classifying events, additional algorithms could be used for tracking the events over time and space as well as evaluating the total probability of the events. The positions of events such as single vehicles or groups of vehicles can be tracked over time using well-known filtering algorithms such as Hidden Markov Models (HMM), Kalman, or particle filtering [16,17]. Using past positions information and current measurements to determine the actual positions provides, in general, higher event detection probability than using only the current measurements.

## 3. Results

We implemented and evaluated two approaches for event detection (see Figure 3): the classic machine learning approach and the deep learning approach. Table 2 summarizes the main design decisions we made according to our system and domain knowledge.

In the following, we present results obtained with classical machine learning and deep learning approaches.

### 3.1. Results with Classical Machine Learning Approach

The ‘classic’ machine learning approach is based on feature extraction and classification. In the following, we describe an application of the classic ML approach in event detection with DAS systems.

#### 3.1.1. Feature Extraction

**Figure 4:**
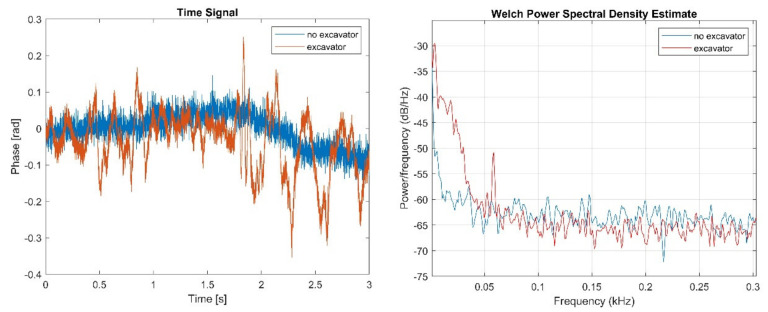
Raw signal: phase of the Rayleigh backscattering light (**left**) and the spectrum of the signal (**right**).

The task of Feature Extraction is to extract from the raw data the features that are representative for the events of interest with enough discriminative power for subsequent classification by machine learning algorithms. An example of a ‘raw’ time signal and the signal spectrum for both the ‘excavator’ and ‘no excavator’ events is presented in Figure 4.

As discussed in Section 2, there are several possible features that can be used for event classification: Time, frequency, and time-frequency features. Among them, the frequency features turned out to be most appropriate because at first, they have clear physical meaning: vibrations are traditionally investigated using frequency analysis, signal sources have usually different spectra, and earth as a transmission media acts as a kind of low pass filter. In addition, [22] recommends the usage of frequency-domain features for event detection along a pipeline using OTDR since they provided good results in the systems presented in their literature review and comprise all the meaningful behaviors of the analyzed signals. Furthermore, it is not clear which time feature can be derived from the signal: amplitudes, duration of oscillations, and number of oscillations, etc. (see Figure 4, left). Time-frequency features such as wavelets also do not have clear physical interpretations and require a larger processing time [11], which might be critical in real-time system deployment. As shown in Figure 4 right, most of the signal spectrum is concentrated below 100 Hz. Consequently, the signal powers at frequencies from 1 to 100 Hz were used as classification features.

**Figure 5:**
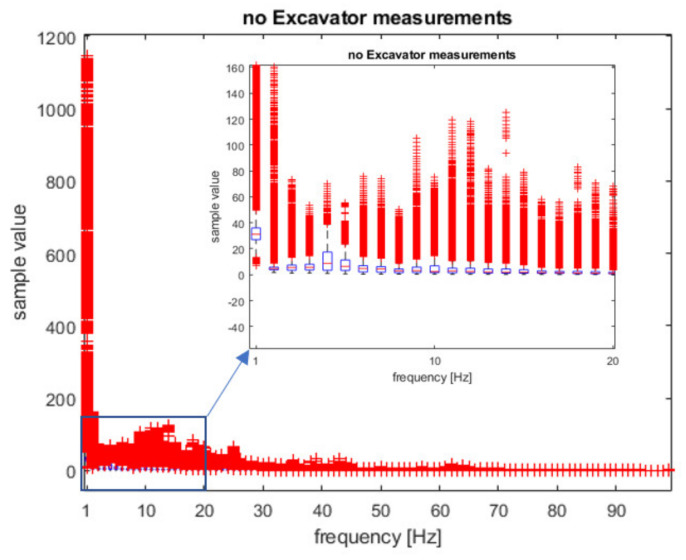
Box plot of the spectrum samples used for classifier training for the ‘no excavator’ event.

**Figure 6:**
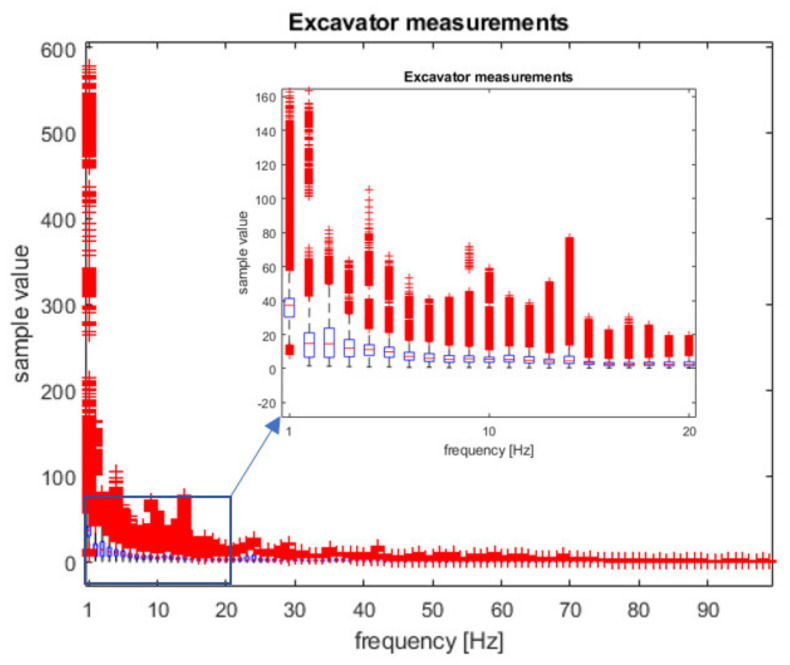
Box plot of the spectrum samples used for classifier training for the ‘excavator’ event.

In Figure 5 and Figure 6, box plots of statistical distributions of spectrum measurements are depicted. These spectrum features are used as the training inputs to ML classifiers to distinguish between two classes of events: ’excavator’ and ‘no excavator’.

As can be seen from Figure 5 and Figure 6, the largest differences between the ‘excavator’ and ‘no excavator’ events are at lower frequencies of 1–10 Hz, especially at the frequencies of 2–4 Hz. We performed a two-sample t-test (using Matlab function ‘ttest2′) for each of the frequencies between 1–100 Hz to estimate which frequencies are relevant for classification. The result was ‘1′ for all frequencies except for the frequency of 1 Hz, i.e., the test rejects the null hypothesis that data comes from normal distributions with equal means and equal but unknown variances (*p*-value < 0.0001). In addition, a Kolmogorov–Smirnov test (function ‘kstest2′) confirmed that the data samples from ‘excavator’ and ‘no excavator’ events come from different probability distributions. We also calculated the effect size of the differences between ‘excavator’ and ‘no excavator’ samples at all 100 frequencies using the Cohen test and obtained 76 frequencies values greater than 0.5, meaning a ‘large effect size’. This means that our data can be used for subsequent classification since they have different means, have a large effect size, and come from different probability distributions. Furthermore, we included in our samples all known interferers such as land machines, wind-wheels, and highways, making our data statistically relevant. We also performed our measurements several times, on different soil types, at different times of the day, and on different days during the week.

**Figure 7:**
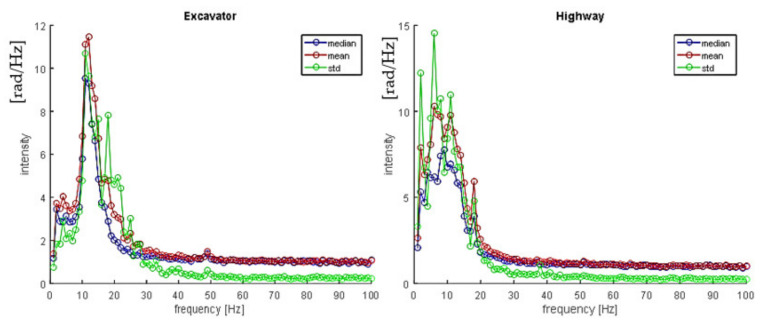
Spectrum statistics of an excavator (**left**) and a highway (**right**).

It is important to stress that all signal characteristics are statistically distributed, with relatively large variance, since signal changes depend on the source type and weight, distance, soil type, and temperature, etc. Furthermore, it is important to exploit differences between the excavator spectrum and possible interferers such as land machines, wind wheels, or highways. For example, Figure 7 represents spectrum statistics from an excavator signal, and a highway signal obtained over 100 sample signals for each signal source.

The high variations in input features such as the spectrum make reliable classification challenging, i.e., distinguishing excavator signals from other interferer signals like a highway. In the next section, we discuss how the classification between ‘excavator’ and ‘no excavator’ events can be performed using classical ML algorithms.

#### 3.1.2. Classification

The following classification algorithms were investigated: k nearest neighbors (k = 1), decision tree (min_samples_leaf = 1, min_samples_split = 2), random forest (max_depth = 100, n_estimators = 100), multi-layer perceptron (MLP) (solver = ‘adam’, alpha = 1 × 10^−5^, hidden_layer_sizes = (20, 2)) and support vector machines (C = 10, gamma = ‘auto’). The optimal parameters of the algorithms were estimated using a grid search (GridSearchCV function from scikit-learn). In Table 3, performance measures of different machine learning algorithms are presented.

For performance evaluation, we used three-fold cross-validation (cross_val_predict function from the scikit-learn library). We also confirmed the results using the test set consisting of 20% of the total data. The performance measures obtained with three-fold cross-validation and verification on the test set are within the 99% confidence interval, which is estimated according to the accuracy, test sample size, and normal error distribution assumption.

**Figure 8:**
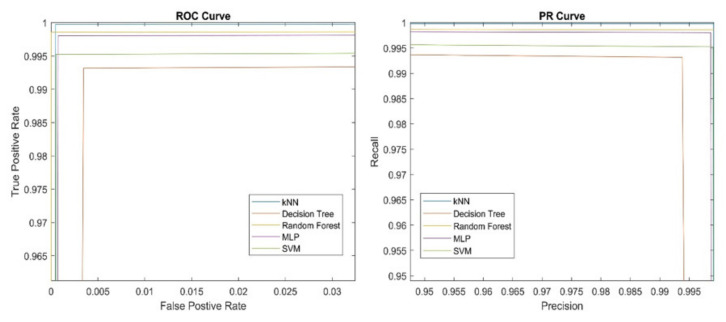
ROC (**left**) and PR (**right**) curves of different classifiers.

As can be seen from Table 3, all classifiers achieved relatively high (above 99%) and similar performances. It is a well-known fact from ML theory that with enough data, almost all classifiers perform well and are almost equally good [31]. This is also confirmed with ROC and PR curves presented in Figure 8 below.

The accuracy of the classifiers is relatively high. According to the comprehensive reviews [22,29], accuracy above 99% is achieved only by sensing much shorter segments (44 m–1 km), and all three classifiers cited in [29] for pipeline monitoring achieved much lower accuracy (55.6%–80.0%). However, the classifiers reviewed in [22] and [29] are mostly multi-event classifiers able to detect different events such as big/small machine hitting, pneumatic hammer, and a plate compactor, while our classifiers distinguish only between two events: ‘excavator digging’ and ‘no excavator digging’. Reference [23] also investigated multiple events: big excavator, small excavator, pneumatic hammer, and plate compactor. The best performances reported in [30] for big excavators were 55.7% (moving) and 31.9% (hitting).

The only significant differences between ML algorithms are in the execution times, especially for test samples, which is relevant for real-time performance (see Table 4). According to Table 4, the lowest execution time was for the decision tree and MLP.

Although the classifier performance, according to Table 3, may appear high, it should be considered that it is only for the signals of a 1-s duration from one virtual sensor of a length of about 10 m. Since a typical DAS unit covers a distance of about 17 km (about 1700 virtual sensors) and a false alarm rate should be lower than once per month, we have a typical problem of rare event detection that often results in a high false alarm rate. For example, if the probability of a false alarm is *p* at one fiber section and within the one-time interval (e.g., one second), then, assuming the independent events in time and space, the number of false alarms is approximately *n × p*, where *n* is the number of fiber sections times the number of time intervals over which the evaluation is completed. To keep the false alarm rate low, we also need a tracking algorithm, as denoted in Figure 3 (left), that records the positions and time steps of the detected events. Consecutive detections of the same event type in close positions are considered the tracking of an event. For example, in addition to the classifier decision (‘an excavator detected’), we can use the following heuristic rule before raising the alarm: an excavator works for a relatively long time (i.e., 90 s instead of 1 s) at approximately the same position (+/− 5 m i.e., one fiber section instead of 1700). Finally, the event probability is evaluated, considering the classification probability of single detections and the track length in seconds. The higher the track length, the more probable is the event. In order to achieve high excavator detection reliability (99.9%) and at a maximum of one false alarm per month, we needed 90 s, i.e., 90 consecutive excavator detections (one per second) at approximately the same position. In this way, the number of possible false alarms can be theoretically reduced from *n* to *n* /(number of fiber section x number of time intervals), i.e., for the approximate factor of 90 × 700 = 153,000 in the case of independent events in time and space. Although in praxis, the possible interference events such as land machines are not independent in time and space, the above calculation shows the theoretical potential of using heuristic rules in reducing the false alarm rate.

The question is whether the detection delay of 90 s can be further reduced by some other approaches to prevent possible intrusions faster. Furthermore, with the above-described classical machine learning approach, we needed almost one second for evaluating one second of real-time data over the length of 17 km; i.e., the processing limit is about 17 km. Increasing the DAS unit range would directly decrease deployment and maintenance costs. It turns out that both a reduction of detection delay and execution time are possible using the deep learning approach.

### 3.2. Deep Learning Approach

**Figure 9:**
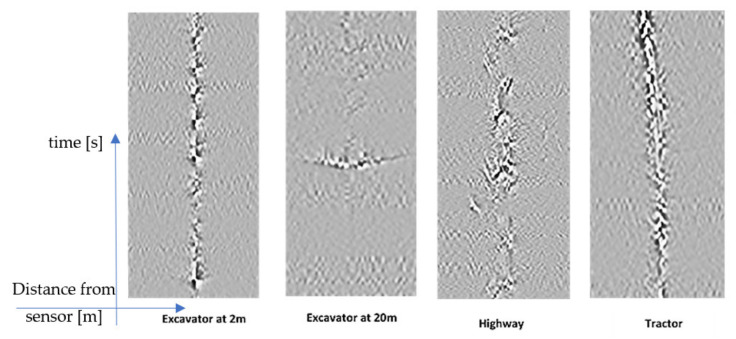
Images of different signal sources. On each image, the horizontal axis represents the distance of the signal source to the DAS sensor in meters and the vertical axis the time in seconds.

Our deep learning approach is based on the idea of converting the sensor signals first to a gray image and then applying a deep neural network to recognize the events in the image. To produce an image from raw sensor data, first, the root mean square (RMS) of 10 ms data (100 samples) for each virtual sensor (10 m) was calculated. The low-pass-filtered RMS values were then processed by a horizontal and vertical Sobel filter to produce a gray image at the end (see the example in Figure 9).

**Figure 10:**
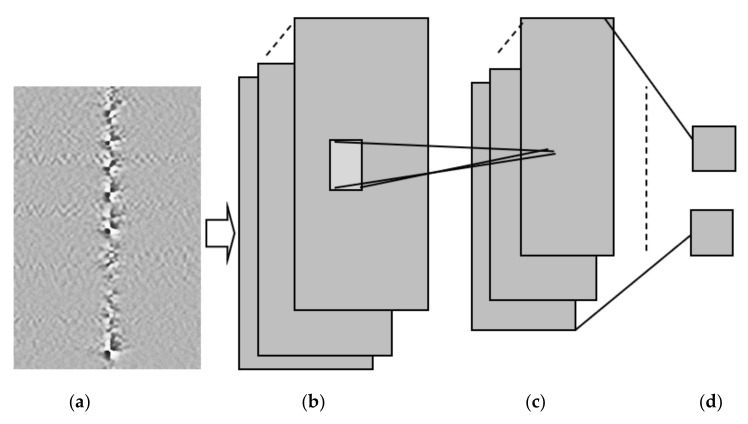
Convolutional neural network (CNN) for excavator detection. (**a**) Input: 50 × 50 Pixels grey (**b**) Convolutional Layer with 5 × 5 filters and 20 (**c**) Max pooling layer with 2 × 2 pooling (**d**) Fully connected.

In the next step, a deep neural network was trained using image samples with an excavator and other signals without an excavator to classify ‘excavator’ vs. ‘no excavator’ events (see Figure 10).

The convolutional neural network (CNN) from Figure 10 consists of the following layers:

- 2D convolutional layer, consisting of 5 × 5 filters with 20 channels (feature maps) in the output of the convolutional layer. The output of each filter *x* is passed to a rectified linear unit that produces max (0, *x*) as its output. The filter is a two-dimensional matrix with parameters trained by a backpropagation algorithm [15]. All filters in one layer share the same set of parameters. The filters at lower layers learn to detect low-level features, such as edges and lines, and the filters at higher layers learn to detect higher-level features, such as shapes.
- 2D max pooling layer, dividing the input into rectangular regions and returning the maximum value of each region. The height and width of the rectangular region (pool size) are both two. This layer creates 2 × 2 pooling regions and returns the maximum of the four elements in each region. Because the stride (step size for moving along the images vertically and horizontally) is also 2 × 2, the pooling regions do not overlap. The role of the pooling layer is signal averaging, i.e., if a part of a shape is detected by one filter and the other part by another filter, the shape could be better detected after pooling these filters together.
- The images are classified into two classes (‘excavator’, ‘no excavator’) by a fully connected output layer that uses the SoftMax function to assign output $y_{k}\;$for each output class *k*:
  (1) $$y_{k}=\frac{ex\;p\left(\theta_{k}^{T}x\right)}{\sum_{j=1}^{K}ex\;p\left(\theta_{j}^{T}x\right)}$$

The parameters were iteratively adapted by training the network using stochastic gradient descent algorithms, with the momentum update of the parameters θ at a time point of *t* + 1, using the values of the parameters at time point t according to the following equations:

(2) $$\theta \left(t+1\right)=\theta \left(t\right)-v$$

We adapted the parameter update using the learning rate α and the momentum constant γ:

(3) $$v\left(t+1\right)=\gamma v\left(t\right)+\alpha \nabla_{\theta }E\left(\theta \right)\;$$

where:

(4) $$E\left(\theta \right)=-\overset{n}{\underset{i=1}{\sum }}\overset{k}{\underset{j=1}{\sum }}t_{ij}\ln y_{j}\left(x_{i},\theta \right)$$

where θ is the parameter vector, *n* is the batch size, *k* is the number of classes (we have k = 2, i.e., two classes: ‘excavator’, ‘no excavator’), $t_{ij}\;$is the indicator that the *i*th sample belongs to the *j*th class, and $y_{j}\left(x_{i},\theta \right)\;$is the output for sample *i*. The output $y_{j}\left(x_{i},\theta \right)$ can be interpreted as the probability that the network associates the *i*th input with class *j*, i.e., P (*t_j_* = 1|*x_i_*).

The network is trained with maximal 50 epochs with random batches of size n = 128 (default value), an initial learning rate α of 0.001, and a momentum constant γ set to 0.9 (default value).

In the classic ML approach, we have to sample each second of new data and track the possibly detected events over several seconds up to a few minutes, using, for example, the Kalman filter. In contrast, our CNN-based approach does not need tracking, since each image already encompasses several tens of seconds up to a few minutes i.e., it already contains a track of an event. Furthermore, the CNN outputs the overall event probability for the whole track, whereas the classic ML approach evaluates the probability of every single detection over the whole track.

The performance of CNN in comparison to the classic ML approach described above is depicted in Table 5. As can be seen from Table 5, both classical ML, like MLP and CNN, achieve similar accuracy, but the execution time of CNN is lower because no feature extraction is needed.

Additionally, in real system deployment, the CNN-based approach outperforms the classic ML approach regarding both the minimum delay required for reliable detection and the execution time. For comparison, we used MLP as the classical ML algorithm with good performance and low execution time (as can be seen from Table 3 and Table 4). CNN needs a six times lower delay and 12 times lower execution time, as can be seen from Table 6. This is because we do not need excessive signal processing for feature extraction, such as in classical machine learning. Furthermore, a CNN does not need a tracking algorithm since the excavator path is captured in the input image. However, the false alarm rate with CNN was also relatively high due to misclassifying interferer signals as land machines or wind wheels vibrations as excavator signals. By combining CNN with simple heuristic rules, we were able to reduce the false alarm rate of the CNN to less than one false alarm per month, as required. We derived the following heuristic rules from the domain knowledge:

- The expected excavator speed is near zero, i.e., the excavator does not change the position over time during the excavation, which helps eliminate land machines as interferers.
- The characteristic frequencies of excavator signals are derived from spectrum analysis of excavator signals, which helps eliminate some interferers, such as wind wheels vibrations, with different spectrums from the excavator.

**Table 6:** Comparison of classic ML approach (MLP) with deep learning approach (CNN) in real-time system deployment in a suburban area over three months.

Algorithms	Number of False Alarms Per Month	Minimum Delay Per Alarm (s)	Execution Time (s)	Max Detection Distance (m)
MLP + heuristic rules	<1	90	60	30
CNN	>10	15	5	10
CNN + heuristic rules	<1	15	5	10

Only the maximum distance where the reliable detection can be achieved is higher in the case of the classic ML-based approach because the finer resolution in the signal strength is possible with raw signals, as used by classic ML, than with images of signals, as used by CNN.

In [22], 98.37% accuracy was reported for the event ‘digging with heavy excavation equipment’ (one of the six distinguishable events). In [25], a classification accuracy of five different event types was reported, ranging between 92.1% for the event ‘digging with a shovel’ and 98.7% for the event ‘walking’. In [26], 94% accuracy was reported using CNN for pipeline protection. We achieved better accuracy than most published papers, but we only distinguished between two events: ‘excavator detected’ and ‘no excavator detected,’ whereas most of the literature investigated more than two events. However, in these works, no reference is made as to when exactly an alarm is generated and how long one should wait before raising the alarm. We tested our system in real-time deployment over three months in a suburban area, but for further verifications, the tests should be provided in different areas and for a longer time.

## 4. Discussion and Conclusions

As shown above, we can achieve the required performance with both classical machine learning algorithms and with a deep neural network. With enough data, almost all machine learning algorithms are equally good [31]. However, using images of the sensor signals and deep neural networks for pattern recognition seems to be a better approach: we could decrease the delay and execution time in comparison to the classic machine learning approach. Since all algorithms must run in real-time to detect possible intrusions, the delay and the execution time are together with accuracy-important performance figures. Decreasing the delay can help the earlier detection of an excavator and prevent early enough possible pipeline damages, and a decreasing execution time enables that the same hardware can cover larger distances of fiber, which reduces costs. Furthermore, we do not need explicit feature selection procedures, such as in the classic machine learning approach, that require a high implementation effort and good domain knowledge. Last, but not least, deep learning CNNs use as inputs the same images that human operators can also evaluate in making their decisions.

We achieved better accuracy than the most published papers, but we only distinguished between two events: ‘excavator’ and ‘no excavator,’ whereas other work distinguished between several events. This might also suggest that in the case of several events, a hierarchical classification could be a better choice, i.e., the first classification might be between ‘excavator’ and ‘no excavator’ events, and then the further classification could be completed within different ‘no excavator’ events such as drilling and hammering, etc. Our system was tested in a real-time deployment of over three months in a suburban area, but for further verifications, the tests should be provided in different areas and for a longer time. However, our main contribution is rather methodological: how to effectively combine classical ML, deep learning, and human knowledge, as outlined below.

Regarding the overall methodology recommendations, it is important to stress that classical and deep learning approaches do not exclude each other; on the contrary, they extend each other, and each realistic project is an interplay between human knowledge, classical machine learning, and the deep learning approach, and is iterative in its nature:

- As the project began, we needed a physical model to decide what, where, when, and how to measure to provide data for machine learning algorithms.
- The classic machine learning approach can be used at the project’s beginning, where little data are available. The classic machine learning approach, combined with human knowledge, provides more insight into the problem domain, i.e., features such as characteristic frequencies relevant for the signal detection, and helps improve physical models of the signal generation. The features extracted from the classic approach can then be used for decisions for which new data might be needed. For example, the interference sources producing similar frequencies as the desired signal are good candidates for collecting new data.
- Deep learning can be used in a later project stage when enough data are collected. Deep neural networks do not need manual feature extractions, which can save a lot of engineering work. Furthermore, deep neural networks can discover unexpected patterns in data that might be unnoticed by a human expert. On the other side, deep neural networks can sometimes discover irrelevant patterns in data and make wrong classifications events with small perturbations of input data. Furthermore, despite all progress in explainable AI, deep neural networks are still a kind of a black box where decisions are not as transparent, as in the case of some classical machine learning techniques such as decision trees [32,33].
- We could achieve the best results by combining the insights from human knowledge about physical models, classical, and deep learning approaches. We used a deep learning network as the core classifier, but we constrained the search domain, only taking into account signals within certain frequencies derived from the physical model and the classical machine learning approach (decision trees). We also eliminated the signals from sources that move too fast to be produced by a standing excavator. This improved accuracy and reduced the probability of false alarms.
- Finally, yet importantly, we need to continue monitoring the system in the deployment. As new data arrive, the system performance may degrade. For example, we noticed that even a few hundred kilometers away, remote earthquakes could cause false alarms. For such cases, automatic updates using information from the internet about interferers, such as earthquakes and construction work, might help. Additionally, including humans in the update loop is needed in order to verify machine/deep learning decisions, define new events, label the data, and improve algorithms.

In this work, we used off-the-shelf CNN without much hyperparameter optimization. In the future, further optimization of the deep neural network can be completed to enable the detection of an excavator at larger distances and the detection of some other interesting events such as manual digging or welding. Transfer learning by the usage of pre-trained neural networks is also an interesting topic for further research.

The method used in this work, converting sensor signals into images and then using CNN for pattern recognition, is a promising approach for optimal sensor fusion in multi-sensor systems. It can be used to detect other intrusion events along the pipeline such as manual digging and tapping, as well as relevant events in other multi-sensor systems. Furthermore, the general methodology of combining human knowledge, classical machine learning, and deep learning is applicable in other science and technology fields such as seismology [34] or medicine [35].

## Figures and Tables

**Table 1:** Hardware and software used in the project.

Component	Characteristics
Hardware	Industry PC: Intel Core i7, 64 GB RAM, 2TB HDD
	Laser: output power: 13 dBm
	Pulse durations: 10/20 ns
	Nominal frequency of 100 MHz
	Phase jitter ≤ ± 10 ps RMS
	Initial frequency accuracy ≤ ± 150 ppm
	Temperature stability ≤ ± 30 ppm
	Aging ≤ ± 15 ppm for the 1st year
	Photodetectors sensitivity: ≥0.85 mA/m
	Data provisioning each second at the rate of 2 kHz
Software	scikit- learn V1.0 (© 2007–2021, scikit-learn developers (BSD License)) library for machine learning Matlab R2019a (The Mathworks, Inc., Natick, MA, USA) for signal processing and machine learning
	Windows 10 (Microsoft Corporation, Redmond, WA, USA) and GNU C for the rest of the software

**Table 2:** Main Design Decisions.

Task	Design Decisions
Data Collection	Total fiber length of 17 km; distance resolution of 10 m.
	Data collection in a suburban area near Vienna, Austria.
	Real-time system evaluations over 3 months.
	DAS: phase-sensitive optical time-domain reflectometer (Φ-OTDR).
	Collect data at different positions along the fiber at different times during the day and different days during the week.
	Collect signals from different interferers: vehicles, land machines, and wind wheels.
	Total of 172,400 signal samples with 100 signal values (one signal value for each frequency from 1 to 100 Hz), with class labels.
	23,020 excavator signals, and 149,440 no excavator signals.
Classical ML (supervised)	Training: 137,968 samples, each with 100 signal values at 1–100 Hz.
	Test: 34,492 samples, each with 100 signal values at 1–100 Hz.
DL CNN (supervised)	Training: 17,054 images (50 × 50 pixels, grey)
	Test: 4264 images (50 × 50 pixels, grey)
Data Evaluation Trigger	Evaluate data from a fiber section when the average signal of the section is above a section-specific threshold.
Feature selection	Use frequency features since they have physical meaning (Earth’s vibrations) and provide the best results in offline simulations.
ML Model Selection	Select the best model using off-line Matlab simulations and N-fold cross-validation.
DNN Selection	Use CNN since they are appropriate for image recognition
Tracking	Use Kalman filter as a widely used standard tracking algorithm

**Table 3:** Performance of different machine learning algorithms.

ML Algorithm	Accuracy	Precision	Recall	F1	AUC	99% Conf. Int.
kNN	99.96%	99.91%	99.98%	0.99	0.99	+/− 0.01%
Decision Tree	99.53%	99.38%	99.32%	0.99	0.99	+/− 0.05%
Random Forest	99.95%	100%	99.86%	0.99	0.99	+/− 0.02%
MLP	99.88%	99.87%	99.80%	0.99	0.99	+/− 0.02%
SVM	99.79%	99.91%	99.52%	0.99	0.99	+/− 0.03%

**Table 4:** Execution time of different ML algorithms on a computer with i7 CPU with 64 GB RAM.

ML Algorithm	Training Time per Instance (μs)	Test Time per Instance (μs)
kNN	-	2048.12
Decision Tree	336.87	0.57
Random Forest	1277.63	16.97
MLP	824.84	0.63
SVM	668.27	358.97

**Table 5:** Comparison of MLP and CNN algorithms performance.

ML Algorithm	Accuracy	99% Conf. Int.	Exec. Time (μs)
MLP + feature extraction	99.88%	+/− 0.02%	554.63
CNN	99.91%	+/− 0.12%	34.33

## Data Availability

Data are provided in the Supplementary File to this manuscript.

## Supplementary Materials

The following are available online at https://www.mdpi.com/article/10.3390/s21227527/s1.
