# Supplementary figures and images for: Event Detection for Distributed Acoustic Sensing: Combining Knowledge-Based, Classical Machine Learning, and Deep Learning Approaches (part 2 of 2)
Source: Sensors (Basel). 2021 Nov 12;21(22):7527. doi: 10.3390/s21227527 (PMC8618866; doi:10.3390/s21227527)

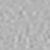

Supplement: Supplementary file 1 [file sensors-21-07527-s001.zip › Data_and_Code_sensors-1424304/DL/DL_Data/noBagger/noBagger_Patch_Wind_1_100_1951.png]

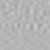

Supplement: Supplementary file 1 [file sensors-21-07527-s001.zip › Data_and_Code_sensors-1424304/DL/DL_Data/noBagger/noBagger_Patch_Wind_1_100_2001.png]

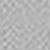

Supplement: Supplementary file 1 [file sensors-21-07527-s001.zip › Data_and_Code_sensors-1424304/DL/DL_Data/noBagger/noBagger_Patch_Wind_1_100_201.png]

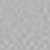

Supplement: Supplementary file 1 [file sensors-21-07527-s001.zip › Data_and_Code_sensors-1424304/DL/DL_Data/noBagger/noBagger_Patch_Wind_1_100_2051.png]

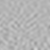

Supplement: Supplementary file 1 [file sensors-21-07527-s001.zip › Data_and_Code_sensors-1424304/DL/DL_Data/noBagger/noBagger_Patch_Wind_1_100_2101.png]

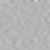

Supplement: Supplementary file 1 [file sensors-21-07527-s001.zip › Data_and_Code_sensors-1424304/DL/DL_Data/noBagger/noBagger_Patch_Wind_1_100_2151.png]

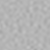

Supplement: Supplementary file 1 [file sensors-21-07527-s001.zip › Data_and_Code_sensors-1424304/DL/DL_Data/noBagger/noBagger_Patch_Wind_1_100_2201.png]

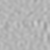

Supplement: Supplementary file 1 [file sensors-21-07527-s001.zip › Data_and_Code_sensors-1424304/DL/DL_Data/noBagger/noBagger_Patch_Wind_1_100_2251.png]

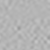

Supplement: Supplementary file 1 [file sensors-21-07527-s001.zip › Data_and_Code_sensors-1424304/DL/DL_Data/noBagger/noBagger_Patch_Wind_1_100_2301.png]

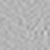

Supplement: Supplementary file 1 [file sensors-21-07527-s001.zip › Data_and_Code_sensors-1424304/DL/DL_Data/noBagger/noBagger_Patch_Wind_1_100_2351.png]

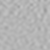

Supplement: Supplementary file 1 [file sensors-21-07527-s001.zip › Data_and_Code_sensors-1424304/DL/DL_Data/noBagger/noBagger_Patch_Wind_1_100_2401.png]

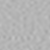

Supplement: Supplementary file 1 [file sensors-21-07527-s001.zip › Data_and_Code_sensors-1424304/DL/DL_Data/noBagger/noBagger_Patch_Wind_1_100_2451.png]

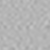

Supplement: Supplementary file 1 [file sensors-21-07527-s001.zip › Data_and_Code_sensors-1424304/DL/DL_Data/noBagger/noBagger_Patch_Wind_1_100_2501.png]

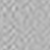

Supplement: Supplementary file 1 [file sensors-21-07527-s001.zip › Data_and_Code_sensors-1424304/DL/DL_Data/noBagger/noBagger_Patch_Wind_1_100_251.png]

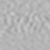

Supplement: Supplementary file 1 [file sensors-21-07527-s001.zip › Data_and_Code_sensors-1424304/DL/DL_Data/noBagger/noBagger_Patch_Wind_1_100_2551.png]

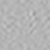

Supplement: Supplementary file 1 [file sensors-21-07527-s001.zip › Data_and_Code_sensors-1424304/DL/DL_Data/noBagger/noBagger_Patch_Wind_1_100_2601.png]

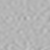

Supplement: Supplementary file 1 [file sensors-21-07527-s001.zip › Data_and_Code_sensors-1424304/DL/DL_Data/noBagger/noBagger_Patch_Wind_1_100_2651.png]

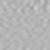

Supplement: Supplementary file 1 [file sensors-21-07527-s001.zip › Data_and_Code_sensors-1424304/DL/DL_Data/noBagger/noBagger_Patch_Wind_1_100_2701.png]

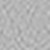

Supplement: Supplementary file 1 [file sensors-21-07527-s001.zip › Data_and_Code_sensors-1424304/DL/DL_Data/noBagger/noBagger_Patch_Wind_1_100_2751.png]

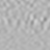

Supplement: Supplementary file 1 [file sensors-21-07527-s001.zip › Data_and_Code_sensors-1424304/DL/DL_Data/noBagger/noBagger_Patch_Wind_1_100_2801.png]

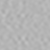

Supplement: Supplementary file 1 [file sensors-21-07527-s001.zip › Data_and_Code_sensors-1424304/DL/DL_Data/noBagger/noBagger_Patch_Wind_1_100_2851.png]

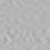

Supplement: Supplementary file 1 [file sensors-21-07527-s001.zip › Data_and_Code_sensors-1424304/DL/DL_Data/noBagger/noBagger_Patch_Wind_1_100_2901.png]

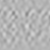

Supplement: Supplementary file 1 [file sensors-21-07527-s001.zip › Data_and_Code_sensors-1424304/DL/DL_Data/noBagger/noBagger_Patch_Wind_1_100_301.png]

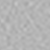

Supplement: Supplementary file 1 [file sensors-21-07527-s001.zip › Data_and_Code_sensors-1424304/DL/DL_Data/noBagger/noBagger_Patch_Wind_1_100_351.png]

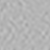

Supplement: Supplementary file 1 [file sensors-21-07527-s001.zip › Data_and_Code_sensors-1424304/DL/DL_Data/noBagger/noBagger_Patch_Wind_1_100_401.png]

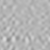

Supplement: Supplementary file 1 [file sensors-21-07527-s001.zip › Data_and_Code_sensors-1424304/DL/DL_Data/noBagger/noBagger_Patch_Wind_1_100_451.png]

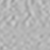

Supplement: Supplementary file 1 [file sensors-21-07527-s001.zip › Data_and_Code_sensors-1424304/DL/DL_Data/noBagger/noBagger_Patch_Wind_1_100_501.png]

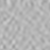

Supplement: Supplementary file 1 [file sensors-21-07527-s001.zip › Data_and_Code_sensors-1424304/DL/DL_Data/noBagger/noBagger_Patch_Wind_1_100_51.png]

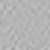

Supplement: Supplementary file 1 [file sensors-21-07527-s001.zip › Data_and_Code_sensors-1424304/DL/DL_Data/noBagger/noBagger_Patch_Wind_1_100_551.png]

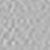

Supplement: Supplementary file 1 [file sensors-21-07527-s001.zip › Data_and_Code_sensors-1424304/DL/DL_Data/noBagger/noBagger_Patch_Wind_1_100_601.png]

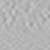

Supplement: Supplementary file 1 [file sensors-21-07527-s001.zip › Data_and_Code_sensors-1424304/DL/DL_Data/noBagger/noBagger_Patch_Wind_1_100_651.png]

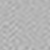

Supplement: Supplementary file 1 [file sensors-21-07527-s001.zip › Data_and_Code_sensors-1424304/DL/DL_Data/noBagger/noBagger_Patch_Wind_1_100_701.png]

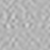

Supplement: Supplementary file 1 [file sensors-21-07527-s001.zip › Data_and_Code_sensors-1424304/DL/DL_Data/noBagger/noBagger_Patch_Wind_1_100_751.png]

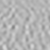

Supplement: Supplementary file 1 [file sensors-21-07527-s001.zip › Data_and_Code_sensors-1424304/DL/DL_Data/noBagger/noBagger_Patch_Wind_1_100_801.png]

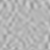

Supplement: Supplementary file 1 [file sensors-21-07527-s001.zip › Data_and_Code_sensors-1424304/DL/DL_Data/noBagger/noBagger_Patch_Wind_1_100_851.png]

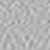

Supplement: Supplementary file 1 [file sensors-21-07527-s001.zip › Data_and_Code_sensors-1424304/DL/DL_Data/noBagger/noBagger_Patch_Wind_1_100_901.png]

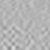

Supplement: Supplementary file 1 [file sensors-21-07527-s001.zip › Data_and_Code_sensors-1424304/DL/DL_Data/noBagger/noBagger_Patch_Wind_1_100_951.png]

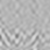

Supplement: Supplementary file 1 [file sensors-21-07527-s001.zip › Data_and_Code_sensors-1424304/DL/DL_Data/noBagger/noBagger_Patch_Wind_1_1000_1.png]

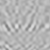

Supplement: Supplementary file 1 [file sensors-21-07527-s001.zip › Data_and_Code_sensors-1424304/DL/DL_Data/noBagger/noBagger_Patch_Wind_1_1000_1001.png]

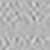

Supplement: Supplementary file 1 [file sensors-21-07527-s001.zip › Data_and_Code_sensors-1424304/DL/DL_Data/noBagger/noBagger_Patch_Wind_1_1000_101.png]

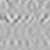

Supplement: Supplementary file 1 [file sensors-21-07527-s001.zip › Data_and_Code_sensors-1424304/DL/DL_Data/noBagger/noBagger_Patch_Wind_1_1000_1051.png]

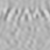

Supplement: Supplementary file 1 [file sensors-21-07527-s001.zip › Data_and_Code_sensors-1424304/DL/DL_Data/noBagger/noBagger_Patch_Wind_1_1000_1101.png]

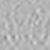

Supplement: Supplementary file 1 [file sensors-21-07527-s001.zip › Data_and_Code_sensors-1424304/DL/DL_Data/noBagger/noBagger_Patch_Wind_1_1000_1151.png]

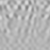

Supplement: Supplementary file 1 [file sensors-21-07527-s001.zip › Data_and_Code_sensors-1424304/DL/DL_Data/noBagger/noBagger_Patch_Wind_1_1000_1201.png]

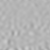

Supplement: Supplementary file 1 [file sensors-21-07527-s001.zip › Data_and_Code_sensors-1424304/DL/DL_Data/noBagger/noBagger_Patch_Wind_1_1000_1251.png]

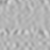

Supplement: Supplementary file 1 [file sensors-21-07527-s001.zip › Data_and_Code_sensors-1424304/DL/DL_Data/noBagger/noBagger_Patch_Wind_1_1000_1301.png]

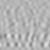

Supplement: Supplementary file 1 [file sensors-21-07527-s001.zip › Data_and_Code_sensors-1424304/DL/DL_Data/noBagger/noBagger_Patch_Wind_1_1000_1351.png]

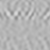

Supplement: Supplementary file 1 [file sensors-21-07527-s001.zip › Data_and_Code_sensors-1424304/DL/DL_Data/noBagger/noBagger_Patch_Wind_1_1000_1401.png]

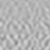

Supplement: Supplementary file 1 [file sensors-21-07527-s001.zip › Data_and_Code_sensors-1424304/DL/DL_Data/noBagger/noBagger_Patch_Wind_1_1000_1451.png]

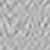

Supplement: Supplementary file 1 [file sensors-21-07527-s001.zip › Data_and_Code_sensors-1424304/DL/DL_Data/noBagger/noBagger_Patch_Wind_1_1000_1501.png]

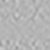

Supplement: Supplementary file 1 [file sensors-21-07527-s001.zip › Data_and_Code_sensors-1424304/DL/DL_Data/noBagger/noBagger_Patch_Wind_1_1000_151.png]

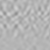

Supplement: Supplementary file 1 [file sensors-21-07527-s001.zip › Data_and_Code_sensors-1424304/DL/DL_Data/noBagger/noBagger_Patch_Wind_1_1000_1551.png]

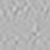

Supplement: Supplementary file 1 [file sensors-21-07527-s001.zip › Data_and_Code_sensors-1424304/DL/DL_Data/noBagger/noBagger_Patch_Wind_1_1000_1601.png]

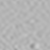

Supplement: Supplementary file 1 [file sensors-21-07527-s001.zip › Data_and_Code_sensors-1424304/DL/DL_Data/noBagger/noBagger_Patch_Wind_1_1000_1651.png]

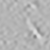

Supplement: Supplementary file 1 [file sensors-21-07527-s001.zip › Data_and_Code_sensors-1424304/DL/DL_Data/noBagger/noBagger_Patch_Wind_1_1000_1701.png]

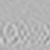

Supplement: Supplementary file 1 [file sensors-21-07527-s001.zip › Data_and_Code_sensors-1424304/DL/DL_Data/noBagger/noBagger_Patch_Wind_1_1000_1751.png]

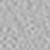

Supplement: Supplementary file 1 [file sensors-21-07527-s001.zip › Data_and_Code_sensors-1424304/DL/DL_Data/noBagger/noBagger_Patch_Wind_1_1000_1801.png]

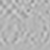

Supplement: Supplementary file 1 [file sensors-21-07527-s001.zip › Data_and_Code_sensors-1424304/DL/DL_Data/noBagger/noBagger_Patch_Wind_1_1000_1851.png]

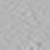

Supplement: Supplementary file 1 [file sensors-21-07527-s001.zip › Data_and_Code_sensors-1424304/DL/DL_Data/noBagger/noBagger_Patch_Wind_1_1000_1901.png]

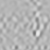

Supplement: Supplementary file 1 [file sensors-21-07527-s001.zip › Data_and_Code_sensors-1424304/DL/DL_Data/noBagger/noBagger_Patch_Wind_1_1000_1951.png]

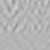

Supplement: Supplementary file 1 [file sensors-21-07527-s001.zip › Data_and_Code_sensors-1424304/DL/DL_Data/noBagger/noBagger_Patch_Wind_1_1000_2001.png]

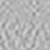

Supplement: Supplementary file 1 [file sensors-21-07527-s001.zip › Data_and_Code_sensors-1424304/DL/DL_Data/noBagger/noBagger_Patch_Wind_1_1000_201.png]

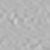

Supplement: Supplementary file 1 [file sensors-21-07527-s001.zip › Data_and_Code_sensors-1424304/DL/DL_Data/noBagger/noBagger_Patch_Wind_1_1000_2051.png]

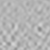

Supplement: Supplementary file 1 [file sensors-21-07527-s001.zip › Data_and_Code_sensors-1424304/DL/DL_Data/noBagger/noBagger_Patch_Wind_1_1000_2101.png]

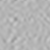

Supplement: Supplementary file 1 [file sensors-21-07527-s001.zip › Data_and_Code_sensors-1424304/DL/DL_Data/noBagger/noBagger_Patch_Wind_1_1000_2151.png]

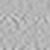

Supplement: Supplementary file 1 [file sensors-21-07527-s001.zip › Data_and_Code_sensors-1424304/DL/DL_Data/noBagger/noBagger_Patch_Wind_1_1000_2201.png]

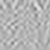

Supplement: Supplementary file 1 [file sensors-21-07527-s001.zip › Data_and_Code_sensors-1424304/DL/DL_Data/noBagger/noBagger_Patch_Wind_1_1000_2251.png]

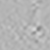

Supplement: Supplementary file 1 [file sensors-21-07527-s001.zip › Data_and_Code_sensors-1424304/DL/DL_Data/noBagger/noBagger_Patch_Wind_1_1000_2301.png]

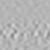

Supplement: Supplementary file 1 [file sensors-21-07527-s001.zip › Data_and_Code_sensors-1424304/DL/DL_Data/noBagger/noBagger_Patch_Wind_1_1000_2351.png]

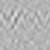

Supplement: Supplementary file 1 [file sensors-21-07527-s001.zip › Data_and_Code_sensors-1424304/DL/DL_Data/noBagger/noBagger_Patch_Wind_1_1000_2401.png]

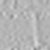

Supplement: Supplementary file 1 [file sensors-21-07527-s001.zip › Data_and_Code_sensors-1424304/DL/DL_Data/noBagger/noBagger_Patch_Wind_1_1000_2451.png]

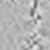

Supplement: Supplementary file 1 [file sensors-21-07527-s001.zip › Data_and_Code_sensors-1424304/DL/DL_Data/noBagger/noBagger_Patch_Wind_1_1000_2501.png]

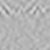

Supplement: Supplementary file 1 [file sensors-21-07527-s001.zip › Data_and_Code_sensors-1424304/DL/DL_Data/noBagger/noBagger_Patch_Wind_1_1000_251.png]

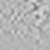

Supplement: Supplementary file 1 [file sensors-21-07527-s001.zip › Data_and_Code_sensors-1424304/DL/DL_Data/noBagger/noBagger_Patch_Wind_1_1000_2551.png]

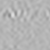

Supplement: Supplementary file 1 [file sensors-21-07527-s001.zip › Data_and_Code_sensors-1424304/DL/DL_Data/noBagger/noBagger_Patch_Wind_1_1000_2601.png]

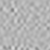

Supplement: Supplementary file 1 [file sensors-21-07527-s001.zip › Data_and_Code_sensors-1424304/DL/DL_Data/noBagger/noBagger_Patch_Wind_1_1000_2651.png]

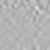

Supplement: Supplementary file 1 [file sensors-21-07527-s001.zip › Data_and_Code_sensors-1424304/DL/DL_Data/noBagger/noBagger_Patch_Wind_1_1000_2701.png]

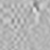

Supplement: Supplementary file 1 [file sensors-21-07527-s001.zip › Data_and_Code_sensors-1424304/DL/DL_Data/noBagger/noBagger_Patch_Wind_1_1000_2751.png]

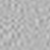

Supplement: Supplementary file 1 [file sensors-21-07527-s001.zip › Data_and_Code_sensors-1424304/DL/DL_Data/noBagger/noBagger_Patch_Wind_1_1000_2801.png]

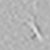

Supplement: Supplementary file 1 [file sensors-21-07527-s001.zip › Data_and_Code_sensors-1424304/DL/DL_Data/noBagger/noBagger_Patch_Wind_1_1000_2851.png]

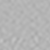

Supplement: Supplementary file 1 [file sensors-21-07527-s001.zip › Data_and_Code_sensors-1424304/DL/DL_Data/noBagger/noBagger_Patch_Wind_1_1000_2901.png]

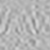

Supplement: Supplementary file 1 [file sensors-21-07527-s001.zip › Data_and_Code_sensors-1424304/DL/DL_Data/noBagger/noBagger_Patch_Wind_1_1000_301.png]

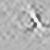

Supplement: Supplementary file 1 [file sensors-21-07527-s001.zip › Data_and_Code_sensors-1424304/DL/DL_Data/noBagger/noBagger_Patch_Wind_1_1000_351.png]

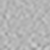

Supplement: Supplementary file 1 [file sensors-21-07527-s001.zip › Data_and_Code_sensors-1424304/DL/DL_Data/noBagger/noBagger_Patch_Wind_1_1000_401.png]

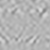

Supplement: Supplementary file 1 [file sensors-21-07527-s001.zip › Data_and_Code_sensors-1424304/DL/DL_Data/noBagger/noBagger_Patch_Wind_1_1000_451.png]

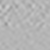

Supplement: Supplementary file 1 [file sensors-21-07527-s001.zip › Data_and_Code_sensors-1424304/DL/DL_Data/noBagger/noBagger_Patch_Wind_1_1000_501.png]

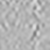

Supplement: Supplementary file 1 [file sensors-21-07527-s001.zip › Data_and_Code_sensors-1424304/DL/DL_Data/noBagger/noBagger_Patch_Wind_1_1000_51.png]

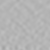

Supplement: Supplementary file 1 [file sensors-21-07527-s001.zip › Data_and_Code_sensors-1424304/DL/DL_Data/noBagger/noBagger_Patch_Wind_1_1000_551.png]

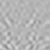

Supplement: Supplementary file 1 [file sensors-21-07527-s001.zip › Data_and_Code_sensors-1424304/DL/DL_Data/noBagger/noBagger_Patch_Wind_1_1000_601.png]

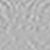

Supplement: Supplementary file 1 [file sensors-21-07527-s001.zip › Data_and_Code_sensors-1424304/DL/DL_Data/noBagger/noBagger_Patch_Wind_1_1000_651.png]

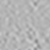

Supplement: Supplementary file 1 [file sensors-21-07527-s001.zip › Data_and_Code_sensors-1424304/DL/DL_Data/noBagger/noBagger_Patch_Wind_1_1000_701.png]

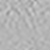

Supplement: Supplementary file 1 [file sensors-21-07527-s001.zip › Data_and_Code_sensors-1424304/DL/DL_Data/noBagger/noBagger_Patch_Wind_1_1000_751.png]

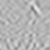

Supplement: Supplementary file 1 [file sensors-21-07527-s001.zip › Data_and_Code_sensors-1424304/DL/DL_Data/noBagger/noBagger_Patch_Wind_1_1000_801.png]

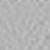

Supplement: Supplementary file 1 [file sensors-21-07527-s001.zip › Data_and_Code_sensors-1424304/DL/DL_Data/noBagger/noBagger_Patch_Wind_1_1000_851.png]

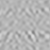

Supplement: Supplementary file 1 [file sensors-21-07527-s001.zip › Data_and_Code_sensors-1424304/DL/DL_Data/noBagger/noBagger_Patch_Wind_1_1000_901.png]

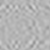

Supplement: Supplementary file 1 [file sensors-21-07527-s001.zip › Data_and_Code_sensors-1424304/DL/DL_Data/noBagger/noBagger_Patch_Wind_1_1000_951.png]

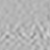

Supplement: Supplementary file 1 [file sensors-21-07527-s001.zip › Data_and_Code_sensors-1424304/DL/DL_Data/noBagger/noBagger_Patch_Wind_1_1100_1.png]
