# Supplementary figures and images for: Event Detection for Distributed Acoustic Sensing: Combining Knowledge-Based, Classical Machine Learning, and Deep Learning Approaches (part 1 of 2)
Source: Sensors (Basel). 2021 Nov 12;21(22):7527. doi: 10.3390/s21227527 (PMC8618866; doi:10.3390/s21227527)

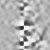

Supplement: Supplementary file 1 [file sensors-21-07527-s001.zip › Data_and_Code_sensors-1424304/DL/DL_Data/Bagger/Bagger_Patch_094245_1_19492000.png]

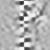

Supplement: Supplementary file 1 [file sensors-21-07527-s001.zip › Data_and_Code_sensors-1424304/DL/DL_Data/Bagger/Bagger_Patch_094245_1_19492025.png]

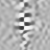

Supplement: Supplementary file 1 [file sensors-21-07527-s001.zip › Data_and_Code_sensors-1424304/DL/DL_Data/Bagger/Bagger_Patch_094245_1_19492050.png]

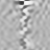

Supplement: Supplementary file 1 [file sensors-21-07527-s001.zip › Data_and_Code_sensors-1424304/DL/DL_Data/Bagger/Bagger_Patch_094245_1_19492075.png]

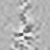

Supplement: Supplementary file 1 [file sensors-21-07527-s001.zip › Data_and_Code_sensors-1424304/DL/DL_Data/Bagger/Bagger_Patch_094245_1_19492100.png]

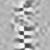

Supplement: Supplementary file 1 [file sensors-21-07527-s001.zip › Data_and_Code_sensors-1424304/DL/DL_Data/Bagger/Bagger_Patch_094245_1_19492125.png]

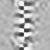

Supplement: Supplementary file 1 [file sensors-21-07527-s001.zip › Data_and_Code_sensors-1424304/DL/DL_Data/Bagger/Bagger_Patch_094245_1_19492150.png]

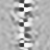

Supplement: Supplementary file 1 [file sensors-21-07527-s001.zip › Data_and_Code_sensors-1424304/DL/DL_Data/Bagger/Bagger_Patch_094245_1_19492175.png]

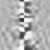

Supplement: Supplementary file 1 [file sensors-21-07527-s001.zip › Data_and_Code_sensors-1424304/DL/DL_Data/Bagger/Bagger_Patch_094245_1_19492200.png]

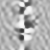

Supplement: Supplementary file 1 [file sensors-21-07527-s001.zip › Data_and_Code_sensors-1424304/DL/DL_Data/Bagger/Bagger_Patch_094245_1_19492225.png]

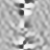

Supplement: Supplementary file 1 [file sensors-21-07527-s001.zip › Data_and_Code_sensors-1424304/DL/DL_Data/Bagger/Bagger_Patch_094245_1_19492250.png]

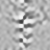

Supplement: Supplementary file 1 [file sensors-21-07527-s001.zip › Data_and_Code_sensors-1424304/DL/DL_Data/Bagger/Bagger_Patch_094245_1_19492275.png]

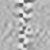

Supplement: Supplementary file 1 [file sensors-21-07527-s001.zip › Data_and_Code_sensors-1424304/DL/DL_Data/Bagger/Bagger_Patch_094245_1_19492300.png]

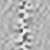

Supplement: Supplementary file 1 [file sensors-21-07527-s001.zip › Data_and_Code_sensors-1424304/DL/DL_Data/Bagger/Bagger_Patch_094245_1_19492325.png]

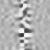

Supplement: Supplementary file 1 [file sensors-21-07527-s001.zip › Data_and_Code_sensors-1424304/DL/DL_Data/Bagger/Bagger_Patch_094245_1_19492350.png]

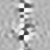

Supplement: Supplementary file 1 [file sensors-21-07527-s001.zip › Data_and_Code_sensors-1424304/DL/DL_Data/Bagger/Bagger_Patch_094245_1_19492375.png]

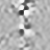

Supplement: Supplementary file 1 [file sensors-21-07527-s001.zip › Data_and_Code_sensors-1424304/DL/DL_Data/Bagger/Bagger_Patch_094245_1_19492400.png]

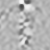

Supplement: Supplementary file 1 [file sensors-21-07527-s001.zip › Data_and_Code_sensors-1424304/DL/DL_Data/Bagger/Bagger_Patch_094245_1_19492425.png]

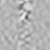

Supplement: Supplementary file 1 [file sensors-21-07527-s001.zip › Data_and_Code_sensors-1424304/DL/DL_Data/Bagger/Bagger_Patch_094245_1_19492450.png]

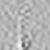

Supplement: Supplementary file 1 [file sensors-21-07527-s001.zip › Data_and_Code_sensors-1424304/DL/DL_Data/Bagger/Bagger_Patch_094245_1_19492475.png]

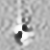

Supplement: Supplementary file 1 [file sensors-21-07527-s001.zip › Data_and_Code_sensors-1424304/DL/DL_Data/Bagger/Bagger_Patch_094245_1_19492500.png]

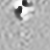

Supplement: Supplementary file 1 [file sensors-21-07527-s001.zip › Data_and_Code_sensors-1424304/DL/DL_Data/Bagger/Bagger_Patch_094245_1_19492525.png]

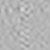

Supplement: Supplementary file 1 [file sensors-21-07527-s001.zip › Data_and_Code_sensors-1424304/DL/DL_Data/Bagger/Bagger_Patch_094245_1_19492550.png]

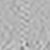

Supplement: Supplementary file 1 [file sensors-21-07527-s001.zip › Data_and_Code_sensors-1424304/DL/DL_Data/Bagger/Bagger_Patch_094245_1_19492575.png]

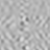

Supplement: Supplementary file 1 [file sensors-21-07527-s001.zip › Data_and_Code_sensors-1424304/DL/DL_Data/Bagger/Bagger_Patch_094245_1_19492600.png]

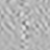

Supplement: Supplementary file 1 [file sensors-21-07527-s001.zip › Data_and_Code_sensors-1424304/DL/DL_Data/Bagger/Bagger_Patch_094245_1_19492625.png]

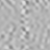

Supplement: Supplementary file 1 [file sensors-21-07527-s001.zip › Data_and_Code_sensors-1424304/DL/DL_Data/Bagger/Bagger_Patch_094245_1_19492650.png]

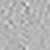

Supplement: Supplementary file 1 [file sensors-21-07527-s001.zip › Data_and_Code_sensors-1424304/DL/DL_Data/Bagger/Bagger_Patch_094245_1_19492675.png]

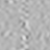

Supplement: Supplementary file 1 [file sensors-21-07527-s001.zip › Data_and_Code_sensors-1424304/DL/DL_Data/Bagger/Bagger_Patch_094245_1_19492700.png]

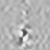

Supplement: Supplementary file 1 [file sensors-21-07527-s001.zip › Data_and_Code_sensors-1424304/DL/DL_Data/Bagger/Bagger_Patch_094245_1_19492725.png]

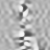

Supplement: Supplementary file 1 [file sensors-21-07527-s001.zip › Data_and_Code_sensors-1424304/DL/DL_Data/Bagger/Bagger_Patch_094245_1_19492750.png]

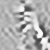

Supplement: Supplementary file 1 [file sensors-21-07527-s001.zip › Data_and_Code_sensors-1424304/DL/DL_Data/Bagger/Bagger_Patch_094245_1_19492775.png]

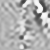

Supplement: Supplementary file 1 [file sensors-21-07527-s001.zip › Data_and_Code_sensors-1424304/DL/DL_Data/Bagger/Bagger_Patch_094245_1_19492800.png]

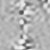

Supplement: Supplementary file 1 [file sensors-21-07527-s001.zip › Data_and_Code_sensors-1424304/DL/DL_Data/Bagger/Bagger_Patch_094245_1_19492825.png]

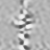

Supplement: Supplementary file 1 [file sensors-21-07527-s001.zip › Data_and_Code_sensors-1424304/DL/DL_Data/Bagger/Bagger_Patch_094245_1_19492850.png]

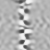

Supplement: Supplementary file 1 [file sensors-21-07527-s001.zip › Data_and_Code_sensors-1424304/DL/DL_Data/Bagger/Bagger_Patch_094245_1_19492875.png]

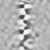

Supplement: Supplementary file 1 [file sensors-21-07527-s001.zip › Data_and_Code_sensors-1424304/DL/DL_Data/Bagger/Bagger_Patch_094245_1_19492900.png]

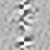

Supplement: Supplementary file 1 [file sensors-21-07527-s001.zip › Data_and_Code_sensors-1424304/DL/DL_Data/Bagger/Bagger_Patch_094245_1_19492925.png]

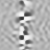

Supplement: Supplementary file 1 [file sensors-21-07527-s001.zip › Data_and_Code_sensors-1424304/DL/DL_Data/Bagger/Bagger_Patch_094245_1_19492950.png]

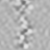

Supplement: Supplementary file 1 [file sensors-21-07527-s001.zip › Data_and_Code_sensors-1424304/DL/DL_Data/Bagger/Bagger_Patch_094745_1_19492000.png]

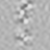

Supplement: Supplementary file 1 [file sensors-21-07527-s001.zip › Data_and_Code_sensors-1424304/DL/DL_Data/Bagger/Bagger_Patch_094745_1_19492025.png]

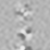

Supplement: Supplementary file 1 [file sensors-21-07527-s001.zip › Data_and_Code_sensors-1424304/DL/DL_Data/Bagger/Bagger_Patch_094745_1_19492050.png]

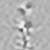

Supplement: Supplementary file 1 [file sensors-21-07527-s001.zip › Data_and_Code_sensors-1424304/DL/DL_Data/Bagger/Bagger_Patch_094745_1_19492075.png]

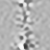

Supplement: Supplementary file 1 [file sensors-21-07527-s001.zip › Data_and_Code_sensors-1424304/DL/DL_Data/Bagger/Bagger_Patch_094745_1_19492100.png]

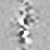

Supplement: Supplementary file 1 [file sensors-21-07527-s001.zip › Data_and_Code_sensors-1424304/DL/DL_Data/Bagger/Bagger_Patch_094745_1_19492125.png]

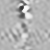

Supplement: Supplementary file 1 [file sensors-21-07527-s001.zip › Data_and_Code_sensors-1424304/DL/DL_Data/Bagger/Bagger_Patch_094745_1_19492150.png]

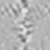

Supplement: Supplementary file 1 [file sensors-21-07527-s001.zip › Data_and_Code_sensors-1424304/DL/DL_Data/Bagger/Bagger_Patch_094745_1_19492175.png]

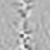

Supplement: Supplementary file 1 [file sensors-21-07527-s001.zip › Data_and_Code_sensors-1424304/DL/DL_Data/Bagger/Bagger_Patch_094745_1_19492200.png]

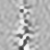

Supplement: Supplementary file 1 [file sensors-21-07527-s001.zip › Data_and_Code_sensors-1424304/DL/DL_Data/Bagger/Bagger_Patch_094745_1_19492225.png]

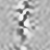

Supplement: Supplementary file 1 [file sensors-21-07527-s001.zip › Data_and_Code_sensors-1424304/DL/DL_Data/Bagger/Bagger_Patch_094745_1_19492250.png]

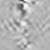

Supplement: Supplementary file 1 [file sensors-21-07527-s001.zip › Data_and_Code_sensors-1424304/DL/DL_Data/Bagger/Bagger_Patch_094745_1_19492275.png]

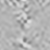

Supplement: Supplementary file 1 [file sensors-21-07527-s001.zip › Data_and_Code_sensors-1424304/DL/DL_Data/Bagger/Bagger_Patch_094745_1_19492300.png]

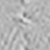

Supplement: Supplementary file 1 [file sensors-21-07527-s001.zip › Data_and_Code_sensors-1424304/DL/DL_Data/Bagger/Bagger_Patch_094745_1_19492325.png]

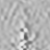

Supplement: Supplementary file 1 [file sensors-21-07527-s001.zip › Data_and_Code_sensors-1424304/DL/DL_Data/Bagger/Bagger_Patch_094745_1_19492350.png]

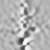

Supplement: Supplementary file 1 [file sensors-21-07527-s001.zip › Data_and_Code_sensors-1424304/DL/DL_Data/Bagger/Bagger_Patch_094745_1_19492375.png]

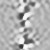

Supplement: Supplementary file 1 [file sensors-21-07527-s001.zip › Data_and_Code_sensors-1424304/DL/DL_Data/Bagger/Bagger_Patch_094745_1_19492400.png]

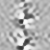

Supplement: Supplementary file 1 [file sensors-21-07527-s001.zip › Data_and_Code_sensors-1424304/DL/DL_Data/Bagger/Bagger_Patch_094745_1_19492425.png]

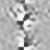

Supplement: Supplementary file 1 [file sensors-21-07527-s001.zip › Data_and_Code_sensors-1424304/DL/DL_Data/Bagger/Bagger_Patch_094745_1_19492450.png]

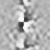

Supplement: Supplementary file 1 [file sensors-21-07527-s001.zip › Data_and_Code_sensors-1424304/DL/DL_Data/Bagger/Bagger_Patch_094745_1_19492475.png]

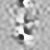

Supplement: Supplementary file 1 [file sensors-21-07527-s001.zip › Data_and_Code_sensors-1424304/DL/DL_Data/Bagger/Bagger_Patch_094745_1_19492500.png]

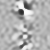

Supplement: Supplementary file 1 [file sensors-21-07527-s001.zip › Data_and_Code_sensors-1424304/DL/DL_Data/Bagger/Bagger_Patch_094745_1_19492525.png]

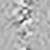

Supplement: Supplementary file 1 [file sensors-21-07527-s001.zip › Data_and_Code_sensors-1424304/DL/DL_Data/Bagger/Bagger_Patch_094745_1_19492550.png]

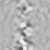

Supplement: Supplementary file 1 [file sensors-21-07527-s001.zip › Data_and_Code_sensors-1424304/DL/DL_Data/Bagger/Bagger_Patch_094745_1_19492575.png]

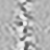

Supplement: Supplementary file 1 [file sensors-21-07527-s001.zip › Data_and_Code_sensors-1424304/DL/DL_Data/Bagger/Bagger_Patch_094745_1_19492600.png]

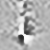

Supplement: Supplementary file 1 [file sensors-21-07527-s001.zip › Data_and_Code_sensors-1424304/DL/DL_Data/Bagger/Bagger_Patch_094745_1_19492625.png]

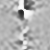

Supplement: Supplementary file 1 [file sensors-21-07527-s001.zip › Data_and_Code_sensors-1424304/DL/DL_Data/Bagger/Bagger_Patch_094745_1_19492650.png]

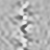

Supplement: Supplementary file 1 [file sensors-21-07527-s001.zip › Data_and_Code_sensors-1424304/DL/DL_Data/Bagger/Bagger_Patch_094745_1_19492675.png]

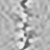

Supplement: Supplementary file 1 [file sensors-21-07527-s001.zip › Data_and_Code_sensors-1424304/DL/DL_Data/Bagger/Bagger_Patch_094745_1_19492700.png]

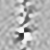

Supplement: Supplementary file 1 [file sensors-21-07527-s001.zip › Data_and_Code_sensors-1424304/DL/DL_Data/Bagger/Bagger_Patch_094745_1_19492725.png]

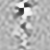

Supplement: Supplementary file 1 [file sensors-21-07527-s001.zip › Data_and_Code_sensors-1424304/DL/DL_Data/Bagger/Bagger_Patch_094745_1_19492750.png]

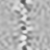

Supplement: Supplementary file 1 [file sensors-21-07527-s001.zip › Data_and_Code_sensors-1424304/DL/DL_Data/Bagger/Bagger_Patch_094745_1_19492775.png]

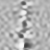

Supplement: Supplementary file 1 [file sensors-21-07527-s001.zip › Data_and_Code_sensors-1424304/DL/DL_Data/Bagger/Bagger_Patch_094745_1_19492800.png]

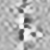

Supplement: Supplementary file 1 [file sensors-21-07527-s001.zip › Data_and_Code_sensors-1424304/DL/DL_Data/Bagger/Bagger_Patch_094745_1_19492825.png]

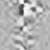

Supplement: Supplementary file 1 [file sensors-21-07527-s001.zip › Data_and_Code_sensors-1424304/DL/DL_Data/Bagger/Bagger_Patch_094745_1_19492850.png]

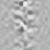

Supplement: Supplementary file 1 [file sensors-21-07527-s001.zip › Data_and_Code_sensors-1424304/DL/DL_Data/Bagger/Bagger_Patch_094745_1_19492875.png]

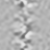

Supplement: Supplementary file 1 [file sensors-21-07527-s001.zip › Data_and_Code_sensors-1424304/DL/DL_Data/Bagger/Bagger_Patch_094745_1_19492900.png]

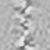

Supplement: Supplementary file 1 [file sensors-21-07527-s001.zip › Data_and_Code_sensors-1424304/DL/DL_Data/Bagger/Bagger_Patch_094745_1_19492925.png]

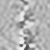

Supplement: Supplementary file 1 [file sensors-21-07527-s001.zip › Data_and_Code_sensors-1424304/DL/DL_Data/Bagger/Bagger_Patch_094745_1_19492950.png]

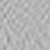

Supplement: Supplementary file 1 [file sensors-21-07527-s001.zip › Data_and_Code_sensors-1424304/DL/DL_Data/noBagger/noBagger_Patch_Wind_1_100_1.png]

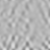

Supplement: Supplementary file 1 [file sensors-21-07527-s001.zip › Data_and_Code_sensors-1424304/DL/DL_Data/noBagger/noBagger_Patch_Wind_1_100_1001.png]

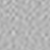

Supplement: Supplementary file 1 [file sensors-21-07527-s001.zip › Data_and_Code_sensors-1424304/DL/DL_Data/noBagger/noBagger_Patch_Wind_1_100_101.png]

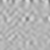

Supplement: Supplementary file 1 [file sensors-21-07527-s001.zip › Data_and_Code_sensors-1424304/DL/DL_Data/noBagger/noBagger_Patch_Wind_1_100_1051.png]

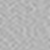

Supplement: Supplementary file 1 [file sensors-21-07527-s001.zip › Data_and_Code_sensors-1424304/DL/DL_Data/noBagger/noBagger_Patch_Wind_1_100_1101.png]

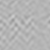

Supplement: Supplementary file 1 [file sensors-21-07527-s001.zip › Data_and_Code_sensors-1424304/DL/DL_Data/noBagger/noBagger_Patch_Wind_1_100_1151.png]

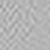

Supplement: Supplementary file 1 [file sensors-21-07527-s001.zip › Data_and_Code_sensors-1424304/DL/DL_Data/noBagger/noBagger_Patch_Wind_1_100_1201.png]

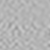

Supplement: Supplementary file 1 [file sensors-21-07527-s001.zip › Data_and_Code_sensors-1424304/DL/DL_Data/noBagger/noBagger_Patch_Wind_1_100_1251.png]

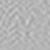

Supplement: Supplementary file 1 [file sensors-21-07527-s001.zip › Data_and_Code_sensors-1424304/DL/DL_Data/noBagger/noBagger_Patch_Wind_1_100_1301.png]

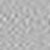

Supplement: Supplementary file 1 [file sensors-21-07527-s001.zip › Data_and_Code_sensors-1424304/DL/DL_Data/noBagger/noBagger_Patch_Wind_1_100_1351.png]

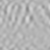

Supplement: Supplementary file 1 [file sensors-21-07527-s001.zip › Data_and_Code_sensors-1424304/DL/DL_Data/noBagger/noBagger_Patch_Wind_1_100_1401.png]

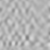

Supplement: Supplementary file 1 [file sensors-21-07527-s001.zip › Data_and_Code_sensors-1424304/DL/DL_Data/noBagger/noBagger_Patch_Wind_1_100_1451.png]

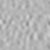

Supplement: Supplementary file 1 [file sensors-21-07527-s001.zip › Data_and_Code_sensors-1424304/DL/DL_Data/noBagger/noBagger_Patch_Wind_1_100_1501.png]

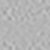

Supplement: Supplementary file 1 [file sensors-21-07527-s001.zip › Data_and_Code_sensors-1424304/DL/DL_Data/noBagger/noBagger_Patch_Wind_1_100_151.png]

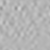

Supplement: Supplementary file 1 [file sensors-21-07527-s001.zip › Data_and_Code_sensors-1424304/DL/DL_Data/noBagger/noBagger_Patch_Wind_1_100_1551.png]

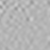

Supplement: Supplementary file 1 [file sensors-21-07527-s001.zip › Data_and_Code_sensors-1424304/DL/DL_Data/noBagger/noBagger_Patch_Wind_1_100_1601.png]

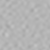

Supplement: Supplementary file 1 [file sensors-21-07527-s001.zip › Data_and_Code_sensors-1424304/DL/DL_Data/noBagger/noBagger_Patch_Wind_1_100_1651.png]

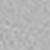

Supplement: Supplementary file 1 [file sensors-21-07527-s001.zip › Data_and_Code_sensors-1424304/DL/DL_Data/noBagger/noBagger_Patch_Wind_1_100_1701.png]

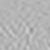

Supplement: Supplementary file 1 [file sensors-21-07527-s001.zip › Data_and_Code_sensors-1424304/DL/DL_Data/noBagger/noBagger_Patch_Wind_1_100_1751.png]

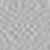

Supplement: Supplementary file 1 [file sensors-21-07527-s001.zip › Data_and_Code_sensors-1424304/DL/DL_Data/noBagger/noBagger_Patch_Wind_1_100_1801.png]

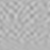

Supplement: Supplementary file 1 [file sensors-21-07527-s001.zip › Data_and_Code_sensors-1424304/DL/DL_Data/noBagger/noBagger_Patch_Wind_1_100_1851.png]

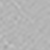

Supplement: Supplementary file 1 [file sensors-21-07527-s001.zip › Data_and_Code_sensors-1424304/DL/DL_Data/noBagger/noBagger_Patch_Wind_1_100_1901.png]
